# Toward optimal disease surveillance with graph-based active learning

**DOI:** 10.1073/pnas.2412424121

**Published:** 2024-12-19

**Authors:** Joseph L.-H. Tsui, Mengyan Zhang, Prathyush Sambaturu, Simon Busch-Moreno, Marc A. Suchard, Oliver G. Pybus, Seth Flaxman, Elizaveta Semenova, Moritz U. G. Kraemer

**Affiliations:** ^a^Department of Biology, University of Oxford, Oxford OX1 3SZ, United Kingdom; ^b^Pandemic Sciences Institute, University of Oxford, Oxford OX3 7DQ, United Kingdom; ^c^Department of Computer Science, University of Oxford, Oxford OX1 3QG, United Kingdom; ^d^Department of Biostatistics, University of California, Los Angeles, CA 90095; ^e^Department of Pathobiology and Population Sciences, Royal Veterinary College, University of London, Hatfield AL9 7TA, United Kingdom; ^f^Department of Epidemiology and Biostatistics, School of Public Health, Imperial College London, London SW7 2AZ, United Kingdom

**Keywords:** disease surveillance, active learning, network dynamics, epidemiology, public health

## Abstract

Tracking the spread of emerging pathogens is critical to the design of timely and effective public health responses. Policymakers face the challenge of allocating finite resources for testing and surveillance across locations, with the goal of maximizing the information obtained about the underlying trends in prevalence and incidence. We model this decision-making process as an iterative node classification problem on an undirected and unweighted graph, in which nodes represent locations and edges represent movement of infectious agents among them. To begin, a single node is randomly selected for testing and determined to be either infected or uninfected. Test feedback is then used to update estimates of the probability of unobserved nodes being infected and to inform the selection of nodes for testing at the next iterations, until certain test budget is exhausted. Using this framework, we evaluate and compare the performance of previously developed active learning policies for node selection, including Node Entropy and Bayesian Active Learning by Disagreement. We explore the performance of these policies under different outbreak scenarios using simulated outbreaks on both synthetic and empirical networks. Further, we propose a policy that considers the distance-weighted average entropy of infection predictions among neighbors of each candidate node. Our proposed policy outperforms existing ones in most outbreak scenarios given small test budgets, highlighting the need to consider an exploration–exploitation trade-off in policy design. Our findings could inform the design of cost-effective surveillance strategies for emerging and endemic pathogens and reduce uncertainties associated with early risk assessments in resource-constrained situations.

## Significance

Infectious diseases primarily spread through human and animal movement networks; study of their transmission dynamics and design of effective interventions requires accurate assessments of the disease distribution at any stage of an outbreak. Disease surveillance has traditionally focused on the rapid detection of infected populations, with little consideration of the overall underlying disease distribution especially when resources are constrained. We address this gap by developing a framework that integrates network structure with the partially observed disease distribution from an outbreak to determine how further testing should be deployed given limited testing resources. Our framework represents an initial step toward the design of more cost-effective local and global surveillance systems for informing public health responses to endemic and emerging pathogens.

Infectious disease surveillance is necessary for managing infectious disease outbreaks, enabling public health authorities to monitor and respond to ongoing disease spread. Notable examples in the past decade include the 2014–2016 West African and 2018–2020 Kivu Ebola virus epidemics, and the COVID-19 pandemic, for which the early detection and continued tracking of the virus’ spread helped to inform the design of interventions including targeted vaccination (1–5), case isolation (6–10), and social distancing (11–14). Without timely and accurate surveillance data, the effectiveness of these interventions would likely have been compromised. For example, travel restrictions targeted at countries where new variants of SARS-CoV-2 were first observed were rendered largely ineffective by delays in case detection and insufficient pathogen sequencing (15, 16). Similarly, the lack of baseline testing prior to the 2015–2016 Zika virus epidemic in the Americas likely contributed to the delay in the identification of the scale of disease spread, thereby allowing the virus to disseminate to new locations before a coordinated response was initiated (17, 18).

Well-documented examples of effective disease surveillance have been limited largely to within-country initiatives [e.g., the Real-time Assessment of Community Transmission (REACT) in the United Kingdom (19) and the National Notifiable Diseases Surveillance System (NNDSS) in the United States (20)], while globally coordinated programs remain rare (21). This can lead to disproportionate or inequitable distributions of testing resources within and between regions or countries, with some locations able to conduct large-scale mass testing for sustained periods of time, while others manage only sparse or sporadic testing (22, 23). One study showed that the intensity of viral genomic sequencing during the COVID-19 pandemic was positively associated with Research & Development expenditures at a country level (24). This likely allowed the virus to continue proliferating undetected in locations with insufficient testing, potentially prolonging local outbreaks.

Previous research on infectious disease surveillance has focused primarily on developing models to identify sentinel sites or subpopulations, with the objective of classifying nodes in networks that could serve as observational units for monitoring disease spread (25–27). Since the COVID-19 pandemic, there has been growing interest in the design of optimal control measures to contain transmission (28), with some studies examining the cost-effectiveness of different strategies for testing and isolation (29–32); one recent study also explored the impact of different air travel regulations on the likelihood of a local epidemic escalating into a global pandemic (33). However, the effectiveness of these interventions depends ultimately on the capacity of local authorities to conduct surveillance and to collectively provide i) timely data of where the disease has been detected (34–36), and ii) an accurate assessment of overall disease distribution (both presence and absence of infections) at any stage of an outbreak—a challenge which, to the best of our knowledge, has received little attention to date.

This study attempts to address this problem; specifically, we consider how testing should be performed across a mobility network, with the objective of providing accurate estimates of where a disease is present, given a fixed budget of testing resources. We hypothesize that the design of an appropriate policy for this task can be formulated as a node classification problem with active learning (AL), where the objective is to select nodes in a partially observed graph for labeling in a manner that maximizes the performance of a model predicting the label of unobserved nodes, while minimizing the amount of labeled data required (37). This motivates the development of an adaptive test deployment framework, which we use to evaluate and compare the performance of previously developed AL policies for infectious disease surveillance. We further propose a policy that takes into consideration graph-based uncertainties, named Selection by Local Entropy (LE), which we show outperforms similar existing policies in most outbreak scenarios and on networks with a diverse range of structural properties, including those commonly found in empirical human mobility networks, especially when test budgets are small.

## Materials and Methods

### Disease Surveillance as a Node Classification Task.

We consider the deployment of a disease surveillance program on a mobility network as a node classification task, in which the mobility network is represented as an undirected and unweighted graph G=(V,E), with nodes $v_{i}\in V$ representing locations, and edges $\left(v_{i},v_{j}\right)\in E$ representing the existence of movement of infectious agents between nodes $v_{i}$ and $v_{j}$. Assuming that there is an underlying distribution of infections resulting from an infectious disease outbreak, the goal of a policymaker (or agent) in this classification task is to predict the presence or absence of the disease (or whether disease prevalence is above or below a certain threshold) at any unobserved node, given the knowledge of the infection status of a subset of nodes in the network.

**Fig. 1.**
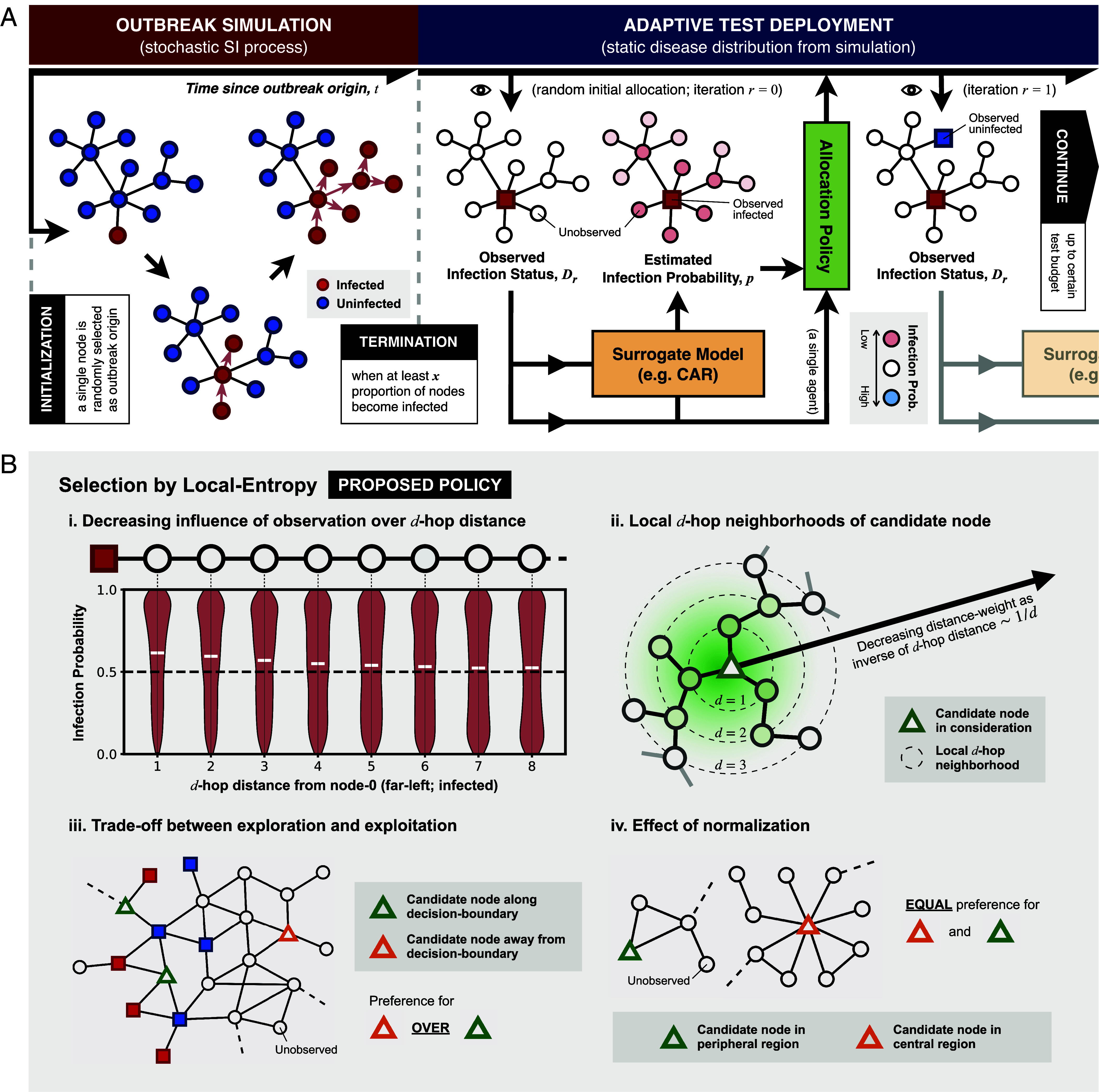
Disease surveillance on a static graph as a node classification task with active learning. (*A*) A schematic illustration of the simulation of infectious disease spread on an undirected and unweighted graph (*Left-hand* side, red compartment), followed by the implementation of a disease surveillance program under an adaptive test deployment framework assuming a static disease distribution (*Right-hand* side, blue compartment). The flow of information/data from one component of the framework to another is represented as arrows. The eye symbol indicates when the underlying disease distribution is queried, thereby revealing the true infection status of a selected node. (*B*) Key concepts behind our proposed policy named Selection by Local Entropy. i) An example showcasing the decreasing influence of an observed node on the estimated infection probability of remaining unobserved nodes in a graph with a chain-like structure. The violin plot shows the posterior distribution of the infection probabilities for the remaining unobserved nodes at different d-hop distances from the observed node on the far-left (node 0); the posterior mean of the probability of each node being infected is indicated by a white horizontal line. The black dashed line indicates an infection probability of 0.5 (i.e., most uncertain). ii) An illustration of the concept of local d-hop neighborhoods, represented by black dashed concentric circles, centered around a candidate node (green triangle). The green shading indicates the distance weight which decreases with increasing d-hop distance from the candidate node following an inverse relationship. iii) An example showcasing the trade-off between exploration and exploitation, with Selection by Local Entropy preferring the selection of the candidate node in the unexplored region (orange triangle) over candidate nodes lying along decision boundaries (green triangles). iv) An example illustrating the effect of normalization by the sum of distance weights over all d-hop neighborhoods (see definition of Local Entropy), resulting in an equal preference for candidate nodes that lie in the peripheral (green triangle) and central (orange triangle) region of a graph.

To generate an underlying disease distribution across the mobility network, we simulate an infectious disease outbreak by modeling its spread as a stochastic Susceptible-Infected (SI) process on graphs, such that transmission can occur only between an infected node and an uninfected node if there is an edge between them. We assume that the outbreak originates from a single, randomly selected node and terminates when a certain proportion (10%, 30%, or 50%) of nodes become infected (Fig. 1*A*, red compartment; see also column 3 in Table 2 and *SI Appendix*, S.1 for further details). Importantly, we assume that the timescale over which transmission occurs is sufficiently longer than the timescale over which testing resources are deployed, such that the resulting disease distribution at the end of the simulated outbreak can be considered as static over the course of the surveillance program (Fig. 1*A*, blue compartment). To indicate the underlying disease distribution, we assign each node $v_{i}$ in the mobility network a binary label $y_{i}\in \left\{0,1\right\}$ to represent its infection status, where $y_{i}=1$ if the node is infected (disease presence) and $y_{i}=0$ if uninfected (disease absence).

Provided that the infection status of a subset of nodes is observed, the infection status of remaining unobserved nodes can then be inferred probabilistically by considering their connections to the observed nodes; we refer to the model that performs this inference as a surrogate model (orange box in Fig. 1*A*). Here, we adopt an approach known as Conditional Autoregressive (CAR) model (38), which estimates the probability that each node is infected (or its posterior distribution under a Bayesian framework) conditional on the infection status of the observed nodes alone (i.e., there are no external data informing the probability estimates except for the observed infection status; *SI Appendix*, S.2 for a detailed description of the model). To assess the degree to which the surrogate model is able to correctly predict the infection status of remaining unobserved nodes given the observed data, we evaluate the Area Under the Receiver Operating Characteristics Curve (AUC) by comparing the infection probability estimates (posterior mean from the CAR model) with the true underlying infection status, where a higher AUC indicates a better predictive performance.

### Test Allocation as an Active Learning Task.

Given a fixed test budget (i.e., a fixed number of tests to be allocated), the predictive performance of the surrogate model will vary depending on which nodes are selected for testing [a task known as AL (37)] and therefore the observed data that are available for model training. To maximize this performance, we consider a number of existing AL policies with a particular focus on those that are adaptive, i.e., policies that select nodes for testing in an iterative fashion until the test budget is exhausted (37). At each iteration, observed data from previous tests are used as input to retrain the surrogate model and to generate infection probability estimates for remaining unobserved nodes; these estimates are then used to guide the selection process at the next iteration, with selection criterion depending on the policy of choice (Fig. 1*A*, blue compartment).

We consider two adaptive AL policies in this study, namely, Node Entropy (NE) (39) and Bayesian Active Learning by Disagreement (BALD) (40). Both of these policies are uncertainty-based, as they select nodes for testing according to where the surrogate model’s predictions are considered to be most uncertain (Table 1 for detailed descriptions of both policies and *SI Appendix*, S.3 for BALD specifically). For comparison, we also consider two nonadaptive, graph-based AL policies, i.e., unobserved nodes are selected for testing by considering only their positions in the network, without using information from previous test iterations (Table 1 for more detailed descriptions).

**Table 1.** Summary of policies considered in this study

Allocation policy	Policy type	Brief description
Least Confidence (LC) (39)	-Uncertainty based -Adaptive	Select the unlabeled node with predicted infection probability (posterior mean) that is closest to 0.5, indicating the least confidence in label prediction.
Node Entropy (NE) (39)		Select the unlabeled node with the highest entropy in its label prediction according to the surrogate model. It can be shown that NE always selects the same node as the policy LC at any iteration (*SI Appendix*, S.4); as a result, only NE is considered hereafter.
Bayesian Active Learning by Disagreement (BALD) (40)		Select the unlabeled node with the highest mutual information between label prediction and posterior from the surrogate model.
Local Entropy (LE) *(our proposed policy)*		Select the unlabeled node with the highest *Local Entropy*, as defined by Eqs. **1**–**3**, with λ=0 (maximal exploration).
Degree Centrality (DC)	-Graph based -Nonadaptive	Select the unlabeled node with the highest degree centrality (most connections).
PageRank Centrality (PC)		Select the unlabeled node with the highest PageRank centrality (41).
Reactive-Infected (RI)	-Benchmark -Adaptive	Select at random an unlabeled node among immediate neighbors of nodes that are known to be infected from previous observations, if available; otherwise, sample randomly from remaining unlabeled nodes.
Random (RAND)	-Benchmark -Nonadaptive	Select an unlabeled node at random.

Abbreviation for each policy is shown in brackets following the policy name. For all policies, random tie-breaking is performed if and when there are multiple candidate nodes given equal preference according to a selection criterion.

**Table 2.** Summary of all experiments conducted in this study

Experiment	Graph(s)	Outbreak scenario(s)
Preliminary *(only uncertainty-based policies are considered)*	Aperiodic lattice graph (with square tiling)	50 random outbreak realizations, with each outbreak terminating when at least 30% of the nodes become infected (I/N=0.3).
Synthetic graphs	Periodic lattice graph (with square tiling) (*graph-based policies are not considered)*	50 random outbreak realizations for each termination condition (I/N=0.1,0.3,0.5); this amounts to a total of 150 random outbreak realization for each graph.
	A random graph generated by the Barabási–Albert model (45), with each node having a minimum of two connections (m=2)	
	A random graph generated by the stochastic block model (46), with low-modularity settings (*SI Appendix*, S.5)	
	A random graph generated by the stochastic block model (46), with high-modularity settings (*SI Appendix*, S.5)	
Empirical human mobility networks	Graphs derived from aggregated mobility data collected from mobile phone users in Italy at the provincial level during March to May, 2020 (47), with thinning thresholds $T_{\mathrm{thinning}}=10\%,15\%,20\%$ (*SI Appendix*, S.6)	
	Graphs derived from global air traffic data collected at the country level during January to March, 2020 (48), with thinning thresholds $T_{\mathrm{thinning}}=2.5\%,5\%,7.5\%$ (*SI Appendix*, S.7)	

### Our Proposed Policy: Selection by Local Entropy (LE).

One potential drawback of using selection criteria based on uncertainty-based metrics alone (as seen in NE and BALD) is that they can lead to a bias toward selecting nodes from regions with highly heterogeneous node labels. In the context of infectious disease surveillance, this can be interpreted as a preference for “exploitation” in an exploration–exploitation trade-off, where exploitation means the selection of nodes that lie along the boundaries between infected and uninfected regions (i.e., decision boundaries) and therefore have highly uncertain infection status predictions despite the availability of data, and “exploration” means the selection of nodes from less observed regions of the graph and therefore with uncertain infection status predictions that are informed by little data (panel iii in Fig. 1*B*). Previous attempts to account for this trade-off have been made, particularly in the context of AL with Graph Neural Network (GNN) models (42), whereby the exploration of less observed regions is encouraged by increasing the probability that a node is selected according to the number of unlabeled neighbors it has (43), or the degree to which the candidate node is representative of its unlabeled neighbors in feature space according to their node attributes (44).

With insights from these previous efforts, we propose here a policy which we refer to as Selection by Local Entropy (LE). This policy evaluates the informativeness of an unlabeled node by taking into account not only the uncertainty in the predicted label of the candidate node itself but also that of surrounding nodes. At a given iteration r, we define the Local Entropy of an unlabeled node $v_{k}$ as a linear combination of the entropy of the label prediction for node $v_{k}$ itself, denoted by $\Omega_{k,r}^{\mathrm{self}}$, and the distance-weighted average entropy of the label predictions for surrounding nodes, denoted by $\Omega_{k,r}^{\mathrm{surr}}$, as follows,

[1] $$\Omega_{k,r}=\lambda \Omega_{k,r}^{\mathrm{self}}+(1-\lambda )\Omega_{k,r}^{\mathrm{surr}},$$

With λ∈[0,1], and

[2] $$\Omega_{k,r}^{\mathrm{self}}=H(v_{k}|D_{r}),$$

[3] $$\Omega_{k,r}^{\mathrm{surr}}=\frac{\sum_{d=1}^{d_{\max}}\sum_{v_{i}\in V(d,v_{k})}H\left(v_{i}|D_{r}\right)/d}{\sum_{d=1}^{d_{\max}}\sum_{v_{i}\in V(d,v_{k})}1/d},$$

where $H(v_{k}|D_{r})$ is the entropy of the label prediction for node $v_{k}$, conditional on the currently observed data $D_{r}=\left\{\left(v_{1},y_{1}\right),\left(v_{2},y_{2}\right),\cdots ,\left(v_{n},y_{n}\right)\right\}$ (*SI Appendix*, S.4 for the formula to calculate the entropy of label prediction in the case of binary classification).

Key insights that motivate the definition of Local Entropy can be summarized as follows:

1. The information that can be gained from the observation of a node is likely to be greater if it is in close proximity to other unlabeled nodes with highly uncertain label predictions [see panel (i) in Fig. 1*B*].
2. The influence that a new observation has on the label predictions for surrounding nodes decays with increasing hopping distance d. This, together with insight (1), motivates the definition of $\Omega_{k,r}^{\mathrm{surr}}$ for a given candidate node $v_{k}$, as the sum of the entropies of the label predictions for all surrounding nodes, with the contribution from nodes in each d-hop neighborhood [denoted by $V(d,v_{k})$] weighted by the inverse of their hopping distance, 1/d (Eq. **3**). This summation extends up to a maximum d-hop distance $d_{\max}$, beyond which the influence of a new observation on the label predictions for unobserved nodes is assumed to be negligible. Altogether, $\Omega_{k,r}^{\mathrm{surr}}$ serves as a proxy measure of the total impact that an observation of a candidate node $v_{k}$ is likely to have on the label predictions for surrounding nodes [see panel (ii) in Fig. 1*B*].
3. This sum, as described in ref. 2, is normalized by the sum of the distance weights (1/d) across all d-hop neighborhoods (up to a hopping distance of $d_{\max}$); this prevents a bias where centrally located nodes would have larger values of $\Omega_{k,r}^{\mathrm{surr}}$, simply due to having more connections. As a result of this normalization, there is an equal preference for nodes in both the peripheral regions (with low centrality) and central regions (with high centrality) of a network, assuming that both regions are equally unexplored [panel (iv) in Fig. 1*B*]
4. The balance between exploration and exploitation (see above) can be fine-tuned by specifying different values of λ. In the case where λ=1, we recover the uncertainty-based policy NE which performs node selection based on node entropy alone.

Note that we set $d_{\max}$ to the graph diameter $d_{G}$ (i.e., the largest geodesic distance between any pair of nodes), in all our following experiments. We also set λ=0 in all subsequent considerations of our proposed policy LE (i.e., maximal exploration).

### Policy Evaluation under Different Network Structures and Outbreak Scenarios.

We conduct three sets of experiments as summarized in Table 2, with each experiment considering a different graph and outbreak scenario. Specifically, we consider synthetic graphs generated by different generative models (column 2 in Table 2) and therefore with different degree distributions and varying levels of community structure and structural disorder. We also consider two empirical human mobility datasets (row 3 in Table 2), from which we derive two unweighted and undirected graphs following a procedure known as graph thinning, where only mobility flows above a certain thinning threshold are preserved (*SI Appendix*, S.6 and S.7 for details).

To explore the impact of different stages of outbreak progression on policy performance, we simulate outbreaks with different termination conditions, as measured by the proportion of nodes that are infected (column 3 in Table 2). For each random outbreak realization on a given network, 25 different nodes are randomly selected as the initial labeled node, with nodes of either infection status being equally likely to be selected; at the beginning of each experiment, the infection status of the same initial labeled node is made available to all agents (with each agent being assigned one of the policies being considered). This is done to account for any variability in policy performance resulting from different initial observations, as well as stochasticity from the Markov chain Monte Carlo (MCMC) inference process and from random tie-breaking whenever two or more candidate nodes are given equal preference by a policy according to its selection criterion.

### Measuring Policy Performance and Test Budget Specifications.

Following the selection of an initial labeled node for a simulated outbreak, as described above, we assess the performance of a given agent (policymaker) at each test iteration by evaluating the AUC, based on a comparison between the current label predictions from the surrogate model (given the available data) and the true infection status of remaining unobserved nodes. The performance of an agent at a given test iteration r can therefore be interpreted as the performance of its designated policy for a given test budget r, assuming no further test deployments.

In each experiment, which considers simulated outbreaks at a specific stage of progression on a given graph, we compare the performance of different policies over a range of test budgets. The maximum test budget is determined by the median number of test iterations required by the Reactive-Infected (RI) policy to identify all infected nodes across all relevant outbreak realizations. Since RI mimics a “contact tracing” approach (Table 1 for a more detailed description of RI), this maximum represents the average minimum number of tests required to identify all infected nodes in a given outbreak scenario. It is therefore only when considering test budgets below this maximum that the objective of accurately predicting the presence or absence of a disease of interest across a mobility network—without complete identification of all infected nodes—may be considered relevant to public health decisions. In all following experiments, we compare the performance of the different policies only at test iterations up to this maximum; full results are presented in *SI Appendix*, Figs. S1 and S2.

## Main Results

### Disease Surveillance on an Aperiodic Regular Lattice Graph.

**Fig. 2.**
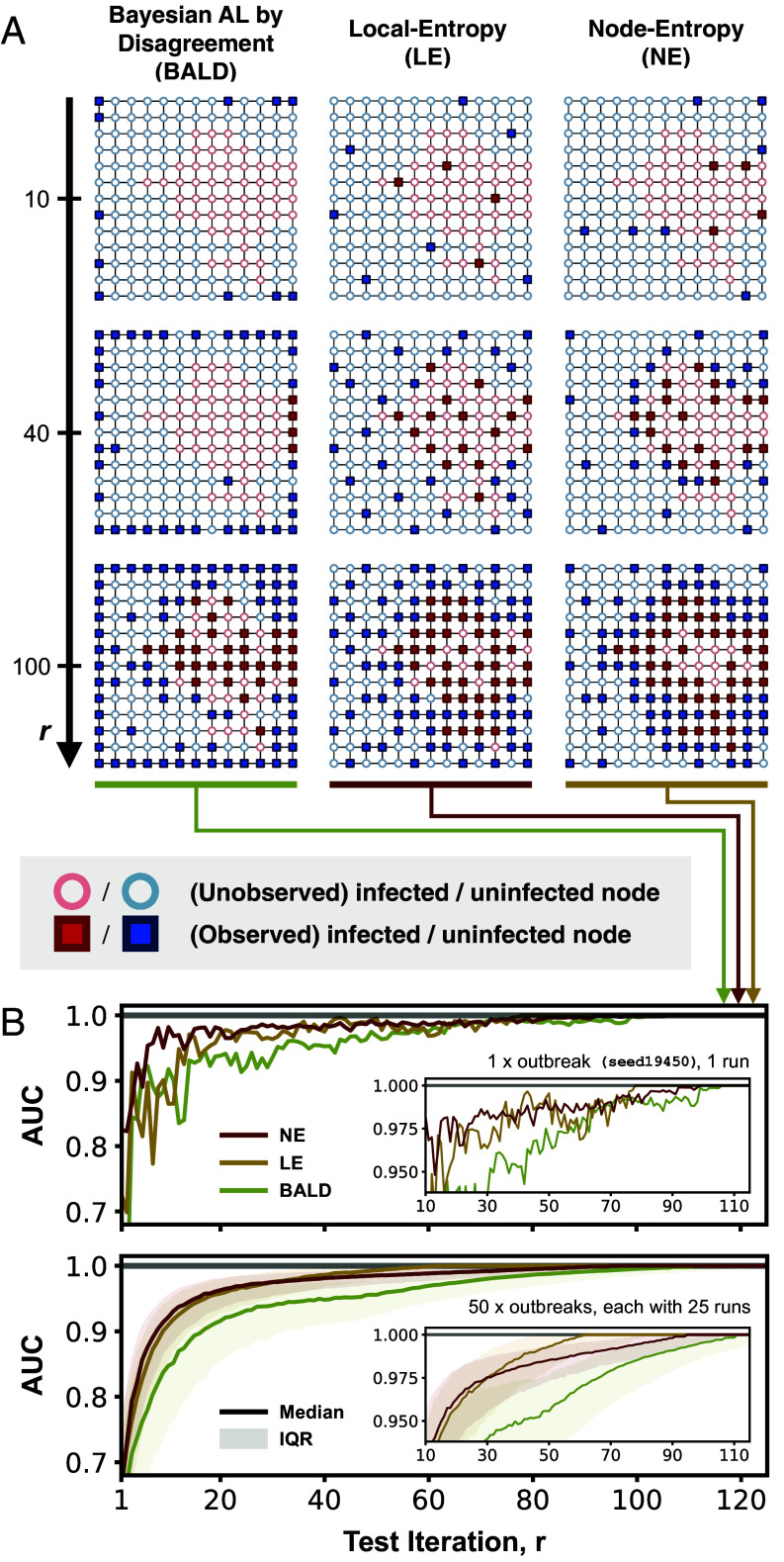
Comparison of Selection by Local Entropy (LE) with existing uncertainty-based policies in the context of simulated outbreaks on an aperiodic lattice graph. (*A*) Test allocation by three selected agents, with each agent assigned a different policy (LE, NE, or BALD). Each square panel shows the distribution of observed (squares) and unobserved (circles) nodes up to a given test iteration (r=10,40,100; as indicated by labels on the *Left*) for a given agent (as indicated by labels at the *Top*). Each node is colored according to its true infection status (red if infected and blue if uninfected, with circles that represent unobserved nodes having a lower opacity). (*B*) The top plot shows the performance of the three selected agents for a single outbreak realization, as measured by the AUC; higher AUC values indicate better performance. The *Bottom* plot shows the performance of LE and the two existing uncertainty-based policies (NE and BALD), each summarized across 1,250 agents (50 outbreak realizations, each with 25 unique initial labeled nodes); the shaded region represents the interquartile range and the solid line represents the median. The *Inset* in each plot shows the same data in the interval 10≤r≤115 on an enlarged scale.

As a preliminary experiment to illustrate the differences between the uncertainty-based policies considered, we evaluate and compare their performance on an aperiodic regular lattice graph with square tiling. We observe that our proposed policy LE on average performs better than both NE and BALD at small numbers of test iterations (r<30; Fig. 2*B*). LE and NE show similar performance between r=30 and r=50; at r>50, however, NE overtakes LE as the best performing policy with an AUC that rapidly approaches 1, while both LE and BALD struggle to attain a perfect AUC. The difference in performance between LE and NE can be understood in the context of the exploration–exploitation trade-off as described above: at small r, LE encourages an even allocation of tests across the graph (exploration), while NE favors regions with highly heterogeneous disease distributions (exploitation) (see columns 2 and 3 in Fig. 2*A*)—this results in a more rapid increase in model performance for LE as r increases. At large r, however, the greater preference for exploitation by NE means that almost all nodes along the decision boundary are sampled; this results in an AUC that rapidly approaches 1. Although LE also shows a preferential selection of nodes close to the decision boundary at large r, it does so at a much slower rate than does NE.

BALD on average performs worse than NE and LE across all test budgets. This is due to its apparent preferential selection of low-degree nodes (either in the corners or along the edges); only at r>40 (at which point no low-degree nodes remain) does BALD exhibit a pattern of test allocation that resembles that of NE. The observed underperformance of BALD is consistent with results from a previous evaluation of existing AL policies for node classification (49), likely explained by the fact that BALD does not consider the graph structure in its formulation (40).

### Disease Surveillance on Synthetic Graphs.

**Fig. 3.**
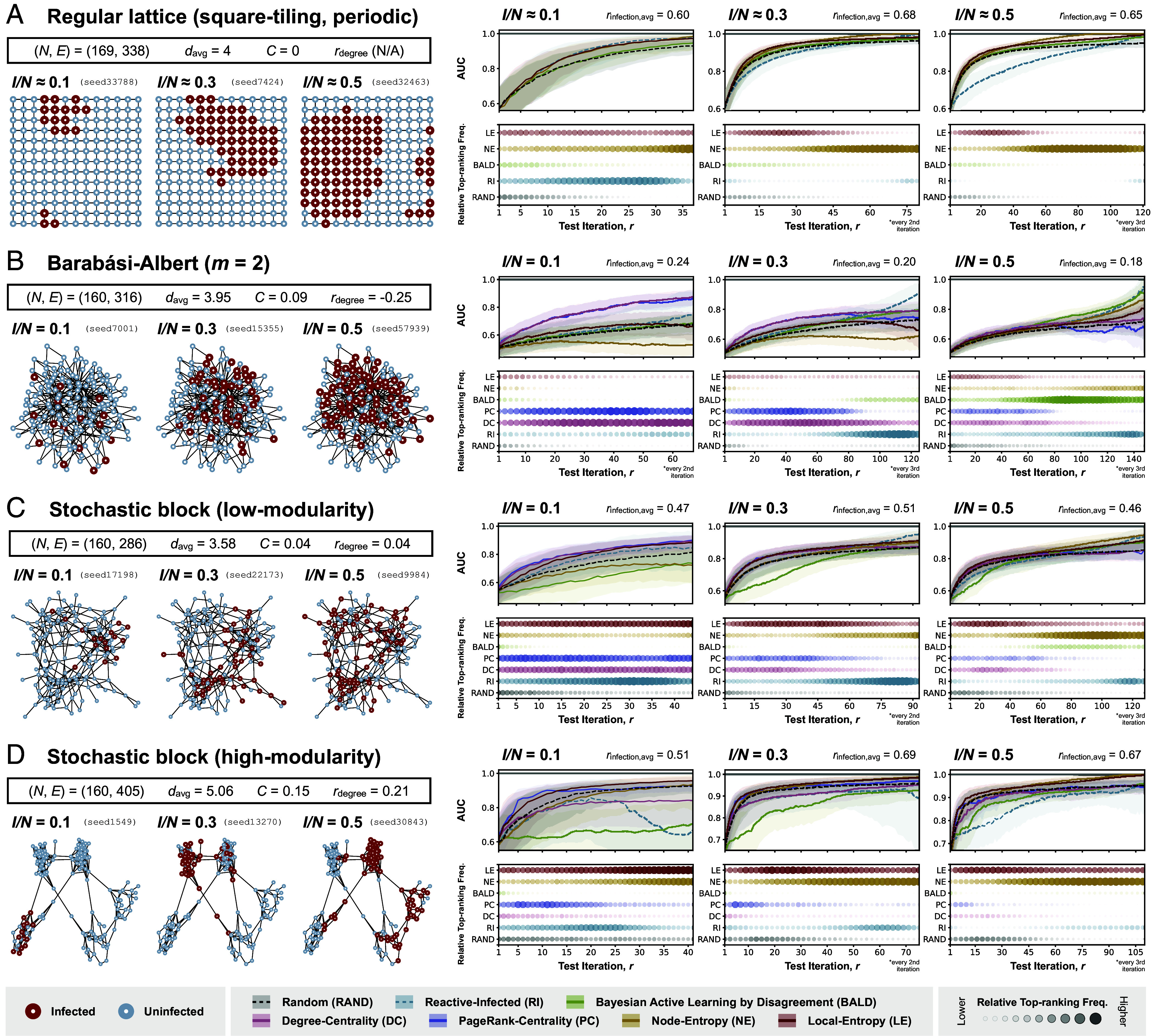
Policy evaluation with simulated outbreaks on synthetic graphs. Each panel (*A*, *B*, *C*, and *D*) corresponds to results from experiments with simulated outbreaks on a different synthetic graph (panel *A*: a periodic regular lattice graph with square tiling; panel *B*: a random graph generated by the Barabási–Albert model, with each node having a minimum of two connections (m=2); (*C*): a random graph generated by the stochastic block model at low-modularity settings; (*D*) a random graph generated by the stochastic block model at high-modularity settings). Summary statistics relevant to the structure of each graph [number of nodes (N), number of edges (E), average node degree ($d_{\mathrm{avg}}$), clustering coefficient (C), and degree assortativity ($r_{\mathrm{degree}}$)] are shown in the *Top-Left* part of each panel. In the *Bottom-Left* part of each panel are visualizations of three selected disease distributions (with their corresponding seeds shown), each at a different stage of outbreak progression as measured by the proportion of nodes infected (I/N=0.1,0.3,0.5); nodes are colored according to their true infection status (red if infected and blue if uninfected). In the *Right* part of each panel, each column shows results from experiments considering disease distributions at a different stage of outbreak progression. The *Top* plot in each column shows the performance of policies considered in the corresponding experiment, as measured by the AUC, with a higher AUC indicating a better performance; the shaded region represents the interquartile range and the solid line represents the median. The *Bottom* plot in each column shows the frequency with which each policy is ranked top according to its AUC at each iteration (or every 2nd or 3rd iteration, where indicated), normalized by the difference between the highest and lowest frequencies across different test budgets in the corresponding experiment (refer to *SI Appendix*, Fig. S5 and Tables S1–S4 for the absolute frequencies); a larger circle with a greater opacity indicates a higher frequency of the policy being ranked top at a given test iteration. The performance of each policy is only shown up to the median number of test iterations required for all infected nodes to be observed among agents assigned to Reactive-Infected (RI) (*SI Appendix*, Fig. S1 for full results).

There are three key observations from our results presented in Fig. 3. First, all policies except for BALD and RI outperform random allocation (RAND) across all outbreak scenarios, especially at large r when the performance of random allocation appears to only increase slowly with increasing r. Given the preferential selection of low-degree nodes by BALD, as mentioned, it is not surprising that BALD only shows comparable performance in the periodic lattice graph which has no degree variation. Second, uncertainty-based policies (NE, BALD, and LE) underperform substantially compared to graph-based heuristics (DC and PC) on the synthetic graph generated by the Barabási–Albert model (hereafter referred to as the BA graph), especially when considering outbreaks at early (I/N=0.1) or intermediate (I/N=0.3) stages, with DC and PC together being ranked top greater than 50% of the time, on average, across all test budgets (*SI Appendix*, Fig. S5 and Table S4). This observation can be explained by considering the infection-assortativity, $r_{\mathrm{infection}}$, which in the context of disease distribution, is a measure of the tendency for two connected nodes to share the same infection status [as has been repeatedly shown in empirical studies that mobility synchronizes epidemics across locations (50); *SI Appendix*, S.8 for definition of infection-assortativity]. Evaluating the average $r_{\mathrm{infection}}$ value across all outbreak realizations on each graph shows that outbreaks on the BA graph have on average the lowest $r_{\mathrm{infection}}$ at 0.20 [compared to 0.64 for the periodic lattice graph, 0.48 and 0.63 for the graphs generated by the stochastic block model (SB graph) with low and high modularity (51), respectively]. A low (but positive) $r_{\mathrm{infection}}$ value indicates a weak tendency for two connected locations to share the same infection status, and therefore a low degree of homophily in the underlying disease distribution. This results in an overall poor predictive performance from the surrogate model, which in turn limits the effectiveness of uncertainty-based policies. In such cases, it may then be advantageous to consider node centrality alone during node selection, especially at small r when there are little data to inform model predictions. Note also that PC tends to perform better than DC—this is not unexpected given that nodes with the most connections are not necessarily the most central in a network.

Finally, we observe generally favorable performance for LE across most of the outbreak scenarios on graphs with a high degree of structural order (unlike the BA graph, as described), especially at small *r*. At larger *r*, however, we again observe superior performance for NE, with AUCs that rapidly approach 1. This can again be explained by the preference for exploitation over exploration by NE, which leads to the complete observation of the decision boundary between infected and uninfected regions given a sufficient number of tests. This is also reflected in the observation that NE is substantially more likely to be ranked top at large r (*Right*-panel in *SI Appendix*, Fig. S5 and Tables S1–S3), compared to LE at small r (partly because of the limited information available when the number of observed nodes is small and therefore smaller distinction in policy performance).

### Disease Surveillance on Empirical Human Mobility Networks.

**Fig. 4.**
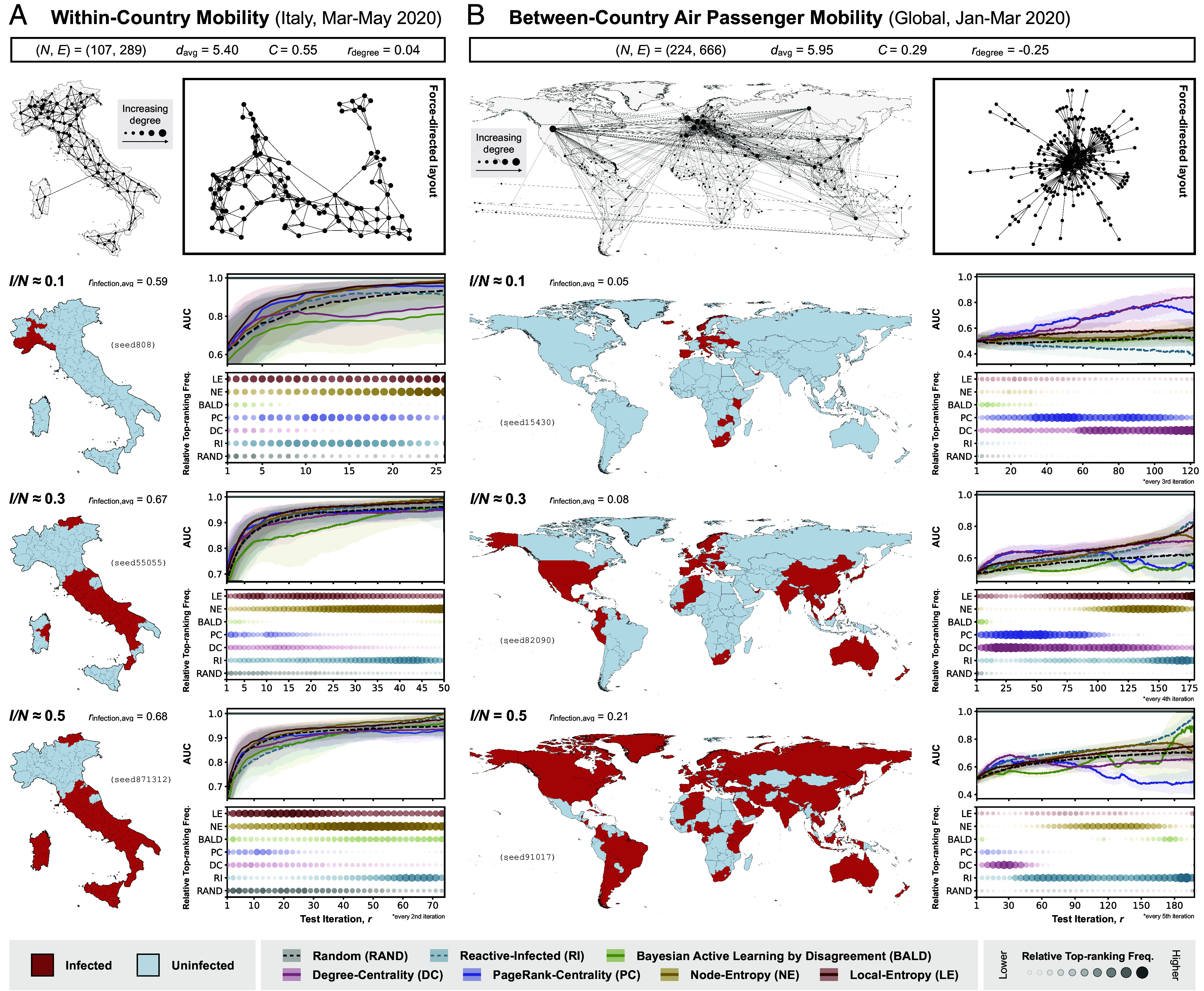
Policy evaluation with simulated outbreaks on graphs derived from empirical human mobility data. Each panel (*A* and *B*) corresponds to results from experiments with simulated outbreaks on a graph derived from a different empirical human mobility dataset (panel *A*: within-country movements from smartphone data collected in Italy at the provincial level between March and May 2020 (39), with thinning threshold $T_{\mathrm{thinning}}=15\%$; panel *B*: between-country human movement from air traffic data collected between January and March 2020 (40), with thinning threshold $T_{\mathrm{thinning}}=5\%$). Summary statistics relevant to the structure of each graph [number of nodes (N), number of edges (E), average node degree ($d_{\mathrm{avg}}$), clustering coefficient (C), and degree assortativity ($r_{\mathrm{degree}}$)] are shown at the top of each panel, followed by (left) a visualization of the graph overlaid on a corresponding map (with the size of each node indicating node degree) and (right) a visualization of the same graph in a force-directed layout. The 2nd to 4th rows of each panel correspond to the different stages of outbreak progression at which the performance of the different policies is evaluated, as measured by the proportion of nodes infected (I/N=0.1,0.3,0.5). In the left part of each row is the visualization of a selected disease distribution (from one of 50) on the corresponding map; tiles are colored according to their true infection status (red if infected and blue if uninfected). In the right part of each panel, each column shows results from experiments considering disease distributions at a different stage of outbreak progression. The *Top* plot in each column shows the performance of policies considered in the corresponding experiment, as measured by the AUC, with a higher AUC indicating a better performance; the shaded region represents the interquartile range and the solid line represents the median. The *Bottom* plot in each column shows the frequency with which each policy is ranked top according to its AUC at each iteration (or every nth iteration, where indicated), normalized by the difference between the highest and lowest frequencies across different test budgets in the corresponding experiment (refer to *SI Appendix*, Fig. S6 and Tables S5 and S6 for the absolute frequencies); a larger circle with a greater opacity indicates a higher frequency of the policy being ranked top at a given test iteration. The performance of each policy is only shown up to the median number of test iterations required for all infected nodes to be observed among agents assigned to Reactive-Infected (RI) (*SI Appendix*, Fig. S2 for full results).

From Fig. 4, it is evident that the two graphs derived from empirical human mobility data have markedly different structural properties. Graph A, generated from aggregated mobility data derived from mobile phone trajectories in Italy at the provincial level (47), shows distinct community structures that closely resemble the SB graphs described in the previous section. In contrast, Graph B, generated from the global air traffic data collected at the country level (48), displays structural properties similar to those of the BA graph. This is consistent with previous studies showing that the global air traffic network has scale-free properties (52, 53) [e.g., both have a negative degree assortativity (Fig. 4*B*), indicating a hub-and-spoke rather than hub-and-hub structure (54)].

We observe that policy performances on Graphs A and B are similar to those from experiments on the SB graphs and BA graph, respectively. Most notably for Graph A, LE again shows rapid increases in model performance given small numbers of test iterations, only to be surpassed by NE at large r, as expected; this observation is consistent across different stages of outbreak progression (*SI Appendix*, Fig. S6). For Graph B, graph-based policies (DC, PC) outperform uncertainty-based policies especially at small r, again consistent with results from experiments on the BA graph. However, the superior performance of these graph-based policies extends only to larger values of r if the outbreak under surveillance is in its early stages (i.e., I/N=0.1); at later stages of outbreak progression, the performance of these policies decreases with further increases in r.

This counterintuitive observation can be explained by considering the changes in the distribution of the decision boundary between the infected and uninfected regions in the graph during a transmission process. At the beginning of an outbreak, nodes that are centrally located are more likely to be infected early on due to their high degree of connectivity. This implies that most of the decision boundary between infected and uninfected regions can be found close to the central nodes, thus explaining the superior performance of graph-based policies which preferentially select nodes with high degree of centrality. As the outbreak progresses, the decision boundary shifts toward the periphery of the graph with the already infected central nodes acting as secondary hubs of the emerging pathogen. This results in a decrease in the performance of graph-based policies, as the central nodes continue to be targeted while the peripheral regions of the graph (where most heterogeneities in the disease distribution lie) remain largely unexplored. Note that a similar drop in the performance of PC (columns 2 and 3 in Fig. 3*B*) at large r during later stages of outbreak progression (I/N=0.3 and I/N=0.5) can also be observed.

The same reasoning can also potentially explain the unexpected superior performance of BALD at large r during later stages of an outbreak on the BA graph (I/N=0.5 in Fig. 3*B*; see also middle- and right-panels in *SI Appendix*, Fig. S5 and Table S4), with most heterogeneities in disease distribution lying in the peripheral regions that are preferentially sampled by BALD. Whereas during the early stages of an outbreak, most heterogeneities in disease distribution are likely to be found in the central regions of a network, therefore resulting in the superior performance of graph-based policies (DC, PC) which target highly connected nodes and RI at small r (I/N=0.1 in Fig. 3 *C* and *D*; see also *Left*-panel in *SI Appendix*, Fig. S5 and Tables S2 and S3), albeit with only modest top-ranking frequencies given the small number of observed nodes. More generally, provided that the number of infected nodes is sufficiently small and that they are confined to a small, local region of the graph, any policy for which there is a high probability of selecting an infected node is likely to perform well compared to other policies, especially when given a small test budget.

## Discussion

In this work, we investigated how a finite amount of testing resources should be allocated across a network of locations connected by mobility, in order to maximize the information gained about the underlying distribution of an infectious disease. We formulate this task as a node classification problem with active learning, with the objective of providing accurate assessment of where the disease is likely to be present or absent given a fixed test budget. We proposed a policy that selects nodes for testing according to a measure of the distance-weighted average entropy of the label predictions in the local neighborhood of a given candidate node. We then evaluated and compared the performance of different policies, including our proposed policy, under a range of different outbreak scenarios and graph structures.

Our results show that in general there is not a single policy that performs optimally across all outbreak scenarios. Instead, the performance of a given policy depends on both the test budget available (relative to the size of the network) and the geometry of the underlying disease distribution, which is in turn determined by network structure and extent of the outbreak. For example, graph-based policies that target central nodes perform better than uncertainty-based policies when the underlying disease spread cannot be modeled with a high degree of accuracy and certainty, as is often the case during early stages of an outbreak when the etiology is unknown. Conversely, uncertainty-based policies are typically more effective in highly ordered networks with well-defined community structures. In particular, with our proposed policy (Selection by Local Entropy) which considers graph-based uncertainties in its selection criterion, we were able to show that more frequent exploration results in better performance given a small test budget, while targeting regions in the network with observed heterogeneous disease distribution (exploitation) is more favorable given a large test budget. Finally, we find that following an approach akin to contact tracing (selecting immediate neighbors of infected nodes) generally leads to inferior performance compared to other policies in terms of characterizing the overall disease distribution. A comprehensive assessment of the overall distribution could potentially allow for a more detailed study of the underlying transmission process (e.g., identifying drivers of spread by iteratively refitting prediction models of disease progression on a network), and provide an opportunity to improve the joint modeling of infectious diseases and sampling more generally.

It should be noted that, while we are able to obtain insights into how different policies are likely to behave under different scenarios, a quantitative assessment of their overall performance—and the extent to which one policy should be recommended over another given any outbreak—requires a more detailed and systematic examination across the various parameter spaces considered, which is beyond the scope of this work. Such assessments are particularly important in comparing the costs and benefits of different policies, especially when little is known about the transmission dynamics of the disease or when the underlying mobility network is unknown (55, 56); future studies should focus on developing appropriate evaluation metrics with consideration of relevant public health contexts and under more realistic model assumptions (see below).

Although we observe consistent results across experiments on both synthetic graphs and empirically derived networks, it is important to interpret these findings in the context of the assumptions made, particularly regarding their generalizability to real-world scenarios. A key limitation of our approach is the assumption that the underlying mobility network can be represented as an undirected and unweighted graph. In reality, mobility networks are highly heterogeneous with mobility fluxes that vary across both regions (e.g., air traffic among European countries versus African countries) and directions (e.g., net inflow of air passengers arriving at tourist destinations during holiday seasons). This limitation is also relevant to infectious diseases with alternative modes of transmission (e.g., sexually transmitted diseases, vector-borne diseases), for which the network capturing the spatial correlation in disease distribution may involve factors other than human movement and cannot be adequately described by an undirected and unweighted graph. For example, in the case of a vector-borne disease, edges in the corresponding network might represent the absence of geographic barriers that prevent vector movement, with edge weights indicating the environmental suitability for vector survival both at the origin/destination location and during transit, which could be time-varying especially for climate-sensitive infectious diseases such as dengue (57–60) and malaria (61–63). Future extensions should consider alternative surrogate models that are able to incorporate these effects when generating label predictions, e.g., GNNs, Gaussian Processes on graphs (64, 65), and spatial mechanistic models that explicitly model the movement of infectious individuals.

Another limitation of this study is the assumption of static disease distributions. This implies that the timescale over which transmission events between locations occur is sufficiently longer than the timescale of test deployment, such that the underlying disease distribution can be treated as static. While this is unlikely to be a realistic assumption for most disease outbreaks—except for some endemic diseases that are more slowly changing in their prevalence, such as HIV/AIDS (66) and Tuberculosis (TB) (67)—it nevertheless allowed us to gain theoretical insights into the various factors one must consider when designing disease surveillance strategies given different network structures and outbreak scenarios. To address this limitation, future work should consider the correlation in infection status not only between nodes but also across time, given either prior assumptions of the underlying transmission dynamics or information from historical transmission events that are inferred to have occurred given the data. In this dynamic setting, it might also be advisable to consider testing multiple locations at once [similar to batch AL (68)], as opposed to only a single location per iteration as presented in our work. Further, future work should also consider the incorporation of external time-series data (e.g., frequency of patients with specific symptoms, rate of hospitalization) and other data types (e.g., pathogen genomic data, wastewater data) that are independent of surveillance efforts and explore how such data can be used to inform test allocation. Finally, we assume here an idealized implementation of disease surveillance, with i) no observational noise (i.e., the true infection status of a selected node is always revealed upon testing), and ii) that each node has equal access to testing resources (i.e., there are no restrictions on which nodes can be selected for testing). However, in practice, i) the infection status of a location could be misclassified due to measurement error or low prevalence, and ii) test deployment at certain locations may be hindered by logistical challenges and limitations in local infrastructures (24). Future studies should consider more realistic assumptions of how testing resources are deployed and their impact on the design of appropriate allocation strategies (e.g., multiple tests might be required at a given location depending on test sensitivity and estimated prevalence).

Our findings are relevant to infectious disease surveillance in resource-constrained settings and in situations where practical challenges render the complete detection of all infected populations unfeasible or cost-inefficient. We propose a flexible and principled approach to evaluating the design and execution of adaptive surveillance strategies with the overall aim of maximizing the information gained from each round of testing. More generally, our adaptive test deployment framework can be extended to consider transmission processes with greater complexities (e.g., SEIR models, spatially explicit semimechanistic models, alternative transmission pathways) and more realistic mobility networks (e.g., as directed and weighted graphs, with time-varying edge weights and node attributes) that are derived from empirical data, and with additional constraints to account for imperfect testing (e.g., observational noise and delay in test feedback, presence of nodes that are inaccessible to surveillance efforts). Applications of our model in real-world contexts could provide the opportunity for more cost-effective and rapid identification and monitoring of pathogens while reducing the uncertainties associated with early risk assessments of infectious diseases.

## Supplementary Material

Appendix 01 (PDF)

## Data Availability

Simulated data and code data have been deposited in Zenodo (69).
